# Assessing Oromotor Capacity in ALS: The Effect of a Fixed-Target Task on Lip Biomechanics

**DOI:** 10.3389/fneur.2019.01288

**Published:** 2019-12-05

**Authors:** Marziye Eshghi, Kaila L. Stipancic, Antje Mefferd, Panying Rong, James D. Berry, Yana Yunusova, Jordan R. Green

**Affiliations:** ^1^Speech and Feeding Disorders Lab, MGH Institute of Health Professions, Boston, MA, United States; ^2^Speech Kinematics and Acoustics Lab, Department of Hearing and Speech Sciences, Vanderbilt University Medical Center, Nashville, TN, United States; ^3^Speech Science and Disorders Lab, Department of Speech-Language-Hearing: Sciences and Disorders, University of Kansas, Lawrence, KS, United States; ^4^Department of Neurology, Massachusetts General Hospital, Boston, MA, United States; ^5^Speech Production Lab, Department of Speech-Language Pathology, University of Toronto, Toronto, ON, Canada

**Keywords:** alternating motion rate, fixed-target tasks, amyotrophic lateral sclerosis, speech kinematics, maximum performance

## Abstract

Alternating motion rate (AMR) is a standard measure often included in neurological examinations to assess orofacial neuromuscular integrity. AMR is typically derived from recordings of patients producing repetitions of a single syllable as fast and clear as possible on one breath. Because the task places high demands on oromotor performance, particularly articulatory speed, AMRs are widely considered to be tests of maximum performance and, therefore, likely to reveal underlying neurologic deficits. Despite decades of widespread use, biomechanical studies have shown that speakers often circumvent the presumed speed challenge of the standard AMR task. Specifically, speakers are likely to manipulate their displacements (movement amplitude) instead of speed because this strategy requires less motor effort. The current study examined the effectiveness of a novel fixed-target paradigm for minimizing the truncation of articulatory excursions and maximizing motor effort. We compared the standard AMR task to that of a fixed-target AMR task and focused specifically on the tasks' potential to detect decrements in lip motor performance in persons with dysarthria due to amyotrophic lateral sclerosis (ALS). Our participants were 14 healthy controls and 17 individuals with ALS. For the standard AMR task, participants were instructed to produce the syllable /bα/ as quickly and accurately as possible on one breath. For the fixed-target AMR task, participants were given the same instructions, but were also required to strike a physical target placed under the jaw during the opening phase of each syllable. Lip kinematic data were obtained using 3D electromagnetic articulography. 16 kinematic features were extracted using an algorithmic approach. Findings revealed that compared to the standard task, the fixed-target AMR task placed increased motor demands on the oromotor system by eliciting larger excursions, faster speeds, and greater spatiotemporal variability. In addition, participants with ALS exhibited limited ability to adapt to the higher articulatory demands of the fixed-target task. Between the two AMR tasks, the maximum speed during the fixed-target task showed a moderate association with the ALSFRS-R bulbar subscore. Employment of both standard and fixed-target AMR tasks is, however, needed for comprehensive assessment of oromotor function and for elucidating profiles of task adaptation.

## Introduction

Oral alternating motion rate (AMR)—elicited during fast repetition of a syllable on a single breath—is a diadochokinetic (DDK) rate measure that, according to Duffy (1), has “the primary value of assessing the speed and regularity of rapid, repetitive articulatory movement” (p. 81). AMR tasks have been used in both research and clinical settings to evaluate neuromuscular integrity by placing high performance demands on the speech subsystems. Impairments in the articulatory, respiratory, phonatory, and resonatory subsystems can result in abnormal AMR task performance, including, but not limited to a shortened task duration, imprecise consonant production, and variable rate of syllable repetition (1).

The AMR task has several well-recognized strengths as an assessment tool of neuromuscular integrity. First, it is time and cost-efficient, non-invasive, and simple to administer. Second, because the task requires only the repetition of short syllables, it can be administered to individuals within a wide age range (2–6), linguistic backgrounds, speech severities (7), and cognitive and linguistic impairments (8, 9). Third, widely-published normative data for AMR tasks across the life-span provide a rich empirical basis for identifying impaired oromotor function (10). Finally, AMR tasks are highly responsive to neuromotor impairments and have previously been used to study neurological disorders such as amyotrophic lateral sclerosis (ALS) (11, 12), multiple sclerosis (13), Parkinson's disease (14–16), and traumatic brain injury (17).

Speech-motor planning, coordination, and control have typically been indexed using acoustic features derived from AMR tasks including the number, duration, and rate of syllable repetitions, intersyllabic gap durations, energy maxima, and voice onset time (17–19). More recently, Rong et al. (12) developed an automated analysis for comprehensively profiling lip and jaw motor performance directly using speech kinematics during AMR tasks. The fine-grained kinematic analyses of AMR tasks have great potential to detect early bulbar function decline in ALS (12, 20).

Despite the widespread use of AMR tasks for early detection and progress monitoring of bulbar signs in ALS, like most measures of maximum performance, AMRs are subject to high inter- and intrasubject variability (10). To maximize the diagnostic efficacy of the assessment, research is needed to understand and subsequently minimize sources of variability related to test administration procedures and participant factors. Although not well-understood, performance on an AMR task can be influenced by a number of factors including familiarity, pre-test practice, instructions, motivation, feedback, age, and sex (2, 3, 10, 21). Perhaps equally concerning is the questionable validity of the task as a maximum performance test. Specifically, contrary to expectations, the task does not elicit maximum movement speeds; rather, many speakers adapt by truncating their articulatory excursions to achieve a fast syllable repetition rate (22–24). The caveat is that although articulatory truncation is an adaptive strategy to economize effort under fast speaking rate conditions, it may mask underlying impairments when used as a compensatory strategy in persons with motor speech disorders.

To minimize opportunities for articulatory truncation, Mefferd et al. (25) developed a fixed-target AMR task to investigate orofacial motor speed capacities in healthy controls and in persons with ALS. Similar approaches have been used to investigate limb motor function in which the speed of reaching movement was derived under a controlled condition by implementing a fixed target (26, 27). The fixed-target AMR task developed by Meffed et al. (25) required talkers to strike a target with the lower jaw during the opening cycle of each syllable. When compared to a standard AMR task, the fixed-target task elicited larger jaw movements, faster jaw speeds, and a more consistent extent of jaw displacement across repetitions in both healthy controls and in talkers with ALS (25). These findings suggested that compared to the standard AMR task, a fixed-target AMR task imposed greater control demands on the oromotor system. Although the study conducted by Mefferd et al. (25) showed that a fixed-target AMR task induced differences between the early-stage bulbar ALS group and healthy controls, only the speed of movement was studied. Therefore, it remains to be determined if kinematic features that can index other aspects of motor performance (e.g., fine motor tuning to spatial, temporal, and spatiotemporal aspects of articulatory biomechanics), are affected by this variant of the AMR task. In addition, fixed-target tasks are not only physically challenging, but also demand greater spatial precision of the lower lip and jaw, which putatively engages extramotor processes such as attention and somatosensation. These additional extramotor processes may engage different neural circuits that otherwise would be absent during habitual speech production. More research is warranted to investigate the putative role of these extramotor processes on speech motor performance.

In this study, we tested the hypothesis that the reduced oromotor capacity due to neurologic disease would negatively impact the overall motor performance during a fixed-target AMR task. In addition, we hypothesized that a fixed-target AMR task would probe both the temporal and spatial aspects of articulatory performance. Our interest was to develop methodologies that can be used to index limitations in oromotor capacity to facilitate improved understanding of the neuromotor basis of speech and swallowing deficits. The aims of the current study were to (1) determine the effect of the fixed-target task on lip kinematics (between-task comparisons) in healthy controls and persons with ALS (between-group comparisons); (2) identify task-related kinematic features that differentiate dysarthric speech due to ALS from the speech of healthy controls; and (3) provide a preliminary evaluation of the clinical validity of the AMR tasks by examining the strength of association between the kinematic measure of “maximum velocity” extracted from the lip and the bulbar subscores on the ALS Functional Rating Scale-Revised (ALSFRS-R). The kinematic measure of “maximum velocity” was of particular interest because the intended purpose of the fixed-target AMR task is to test the velocity generating capacity of the lip by minimizing opportunities for articulatory truncation.

## Materials and Methods

### Participants

Participants included 14 healthy controls (8 males and 6 females, mean age = 62.23, SD = 9.04) and 17 patients with ALS (8 males and 9 females, mean age = 53.65, SD = 7.08). Healthy controls reported no history of speech, language, hearing, or neurological disorders. All participants were native speakers of American English, passed a hearing screening, and had cognition adequate to follow the task instructions. Of 17 participants with ALS, the ALS Cognitive Behavioral Screen (ALS CBS (28)) scores were available for 10. The test yields a total cognitive score ranging from 0 to 20. The mean ALS CBS score for these participants was 15.7 (SD = 2.3). A score of 16 has been identified as the optimal cognitive cut-off score to differentiate intact cognition from potential cognitive impairment (28), 6 participants demonstrated cognitive scores above this threshold (mean = 17.33, SD = 0.52, ranging from 17 to 18), and four participants demonstrated cognitive scores below that (mean = 13.25, SD = 1.5, ranging from 12 to 15). Participants with ALS had been diagnosed with ALS by a neurologist based on the El Escorial criteria (29) and were required to have no history of speech, language, hearing, or other neurological disorders. Participants with ALS varied in the site of onset (11 with spinal onset, 4 with bulbar onset, and 2 unknown). Of 17 participants with ALS, the ALSFRS-R (30) scores were available for 13 participants. The mean ALSFRS-R total score was 30.23 (SD = 7.21) and the mean bulbar sub-score was 8.85 (SD = 1.63). Table 1 displays the scores of individual items on the ALSFRS-R self-rated by the participants with ALS.

**Table 1:** ALSFRS-R scores of individual items in patients with ALS.

**Subjects**	**Q1: speech**	**Q2: salivation**	**Q3: swallowing**	**Q4: handwriting**	**Q5: cutting food**	**Q6: dressing and hygiene**	**Q7: turning in bed**	**Q8: walking**	**Q9: climbing stairs**	**Q10a: breathing**	**Q10b: breathing**	**Q10c: breathing**	**ALSFRS-R** **Total score**	**ALSFRS-R** bulbar subscores^*^
ALS_1	3	3	3	3	2	1	1	1	0	2	2	4	25	9
ALS_2	3	3	3	0	0	0	0	1	0	4	3	4	21	9
ALS_3	3	4	3	3	4	4	3	3	1	4	3	4	39	10
ALS_4	2	4	2	4	4	4	4	4	2	3	4	2	39	8
ALS_5	3	4	3	0	0	0	0	0	0	4	4	4	22	10
ALS_6	2	3	3	2	1	1	3	3	3	4	4	4	33	8
ALS_7	3	4	3	3	1	1	1	1	0	2	4	4	27	10
ALS_8	4	4	4	2	1	1	1	2	0	2	3	2	26	12
ALS_9	2	2	3	3	3	3	3	3	3	3	4	2	34	7
ALS_10	1	3	3	4	2	3	3	4	3	3	4	4	37	7
ALS_11	3	3	3	3	1	2	2	3	3	3	3	4	33	9
ALS_12	3	4	3	2	3	3	3	3	3	3	4	4	38	10
ALS_13	2	2	2	0	0	0	1	0	0	4	4	4	19	6

Each task is rated on a five-point scale from 0 = extreme difficulty, to 4 = normal ability.

* *ALSFRS-R bulbar subscores is the summation of the speech, salivation, and swallowing items*.

Participants with ALS were identified to have bulbar symptoms because they exhibited a habitual speaking rate of slower than 150 words per min (w/m) following Rong et al. (31). Speaking rate was used as a criterion to identify these participants because previous research has demonstrated that the decline of speaking rate precedes, and has a faster rate of decline, than speech intelligibility during the early stage of ALS (32, 33). The mean speaking rate for the participants with ALS was 114.07 w/m (ranged from 54.61 to 146.02 w/m, SD = 28.07) and for healthy controls was 188.20 w/m (ranged from 155.76 to 216.26 w/m, SD = 18.22). Mean speech intelligibility scores for participants with ALS was 88.50% (ranged from 23.64 to 100%, SD = 22.23) and for healthy controls was 99.94% (ranged from 99.09 to 100%, SD = 0.24). Speaking rate and speech intelligibility were measured using the procedures described in the Speech Intelligibility Test (SIT) manual (34). This study was approved by the Partners Healthcare Institutional Review Board (IRB) and all participants provided written, informed consent to participate in the study.

### Procedures

For the standard AMR task, participants were asked to take a deep breath and to produce the syllable /bα/ as quickly and accurately as possible until they ran out of breath. For the fixed-target AMR task, participants were given the same instructions but additionally, the experimenter held a target under the chin of the participant. The target was a stick with a blunt plastic tip. The distance between the target and the lower jaw was determined by asking participants to produce the syllable /bα/ and hold their mouths open for a few seconds. The target was positioned so that it touched the underside of the chin during maximum jaw opening, which occurs during the vowel. To stabilize the target position, the experimenters stood behind the participants with their left hand holding the target in position while stabilizing it against the participants' sternum. The experimenters' right hand was then used to stabilize the participants' head for the purpose of minimizing forward head rotation. Participants were instructed to hit the stick with each production. If participants failed to hit the target, they were encouraged to continue and to attempt to hit the target on the next production. Participants practiced the task before recording began.

### Kinematic Recordings

Movements of the articulators during the AMR tasks were captured using a 3D electromagnetic articulograph (Wave; Northern Digital, Inc.). Following procedures described by Rong et al. (12), the 3D Euclidean distance between sensors attached to the midline of the upper and lower lip was derived during each of the AMR tasks, and subsequently low-passed filtered at 15 Hz. These movement signals were used in the following data analyses.

### Extraction of Kinematic Features

A semi-automatic MATLAB algorithm was used to quantify features of lip performance from the 3D lip movement time-series. This algorithm derives features that probe various aspects of oromotor performance including spatial, temporal, and spatiotemporal characteristics of articulatory movement as well as the overall motor performance (12). The algorithm automatically segmented the opening and closing phases of the lip distance traces and identified cycles with large spatial or temporal deviations from the average pattern across cycles for manual inclusion or exclusion of the deviant cycles (12). Based on the lip movement traces, the algorithm extracted 22 lip movement kinematic features during the AMR tasks. Of the 22 output kinematic features, only 16 features were selected to study because they were considered to be clinically interpretable. All 16 features were included in our analysis because (1) there was no a priori basis for excluding variables and (2) prior research will benefit from identifying the best subset of features for staging bulbar motor involvement.

Table 2 displays the features of interest used in this study. Spatial, temporal, and spatiotemporal features as well as the features of overall motor performance are consistently color-coded in blue, orange, green, and pink, respectively in Table 2 and further illustrations hereafter.

**Table 2:** Kinematic features extracted from the upper and lower lip movement during AMR tasks.

	**Measures**	**Description**
Spatial features	Slp_1 (mm)	Slope of the linear regression lines representing peaks of lip movement throughout all AMR cycles
	Slp_2 (mm)	Slope of linear regression lines representing troughs of lip movement throughout all AMR cycles
	Sse1 (mm)	Lip opening gestural variability: Root_mean_square of residuals of the slope of regression line for peaks of lip movement
	Sse2 (mm)	Lip closing gestural variability: Root_mean_square of residuals of the slope of regression line for troughs of lip movement
	Scanning_d1 (mm)	Mean of absolute differences of peaks (i.e., lip opening) in consecutive cycles
	Scanning_d2 (mm)	Mean of absolute differences of troughs (i.e., lip closing) in consecutive cycles
	max_open (mm)	Maximum lip opening distance
	max_close (mm)	Maximum lip closing distance
Temporal features	Tsd (s)	Standard deviation of cycle duration
	Jitter (s)	Mean of absolute differences of duration in consecutive cycles
	F (cycles/s)	Frequency of syllable repetitions
Spatiotemporal features	Sti	Spatiotemporal variability index
	d_dtw	Dynamic time warping distance: index of dissimilarity between lip distance time series and a sine wave with the same frequency and average amplitude
	Max_vel (mm/s)	Maximum velocity of lip movement across all cycles
Overall performance	Dur (s)	Total duration of the AMR sequence
	Ncyc (cycles)	Total number of cycles in the AMR sequence

**Figure 1:**
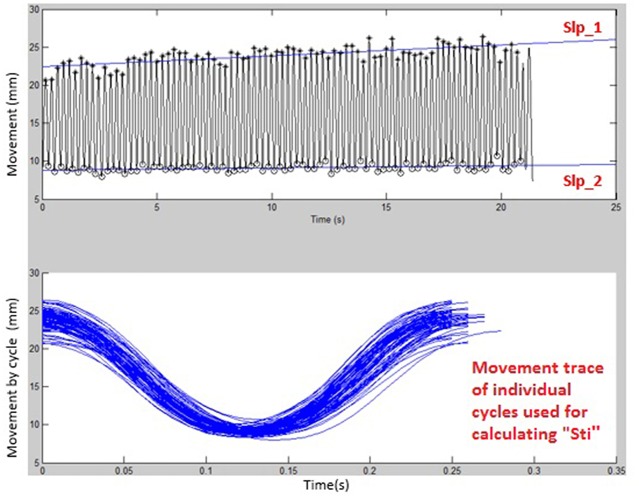
Kinematic features of Slp_1, Slp_2, and individual cycles (from which the Sti feature was calculated) extracted from lip movements of one healthy control participant during a standard AMR task.

**Figure 2:**
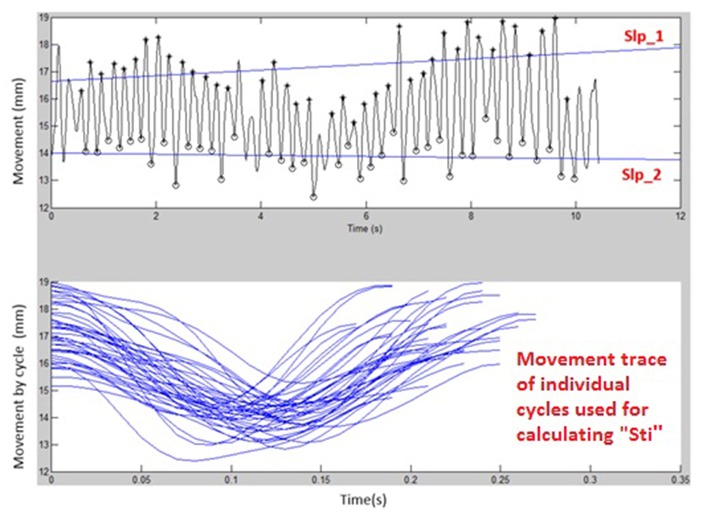
Kinematic features of Slp_1, Slp_2, and individual cycles (from which the Sti feature was calculated) extracted from lip movements of one participant with ALS during a standard AMR task.

Figures 1, 2 display examples of MATLAB plots for Slp_1, Slp_2, and Sti features extracted from the /bα/ syllables produced by a healthy control participant and a participant with ALS during the standard AMR task. Because the rest of features were mainly mathematic notations, corresponding plots of other features were not produced.

## Statistical Analysis

Data were statistically analyzed using R version i386 3.5.3 (35) to examine task and group differences and *p*-values were compared against the 0.05 significance level selected a priori. Paired sample *t*-tests were used to examine the within-group task related differences and independent samples *t*-tests were used to compare the motor performance of groups across standard and fixed-target AMR tasks. Additionally, linear regression analyses were performed to examine the numerical association between the Max_vel in both types of AMR tasks (i.e., standard and fixed-target tasks) and the ALSFRS-R bulbar subscores.

## Results

### Within-Group Comparison of Standard and Fixed-Target AMR Tasks

Healthy controls showed statistically significant differences between the standard and fixed-target AMR tasks for the majority of kinematic features of lip movement (spatial, temporal, spatiotemporal, and overall performance) (*p* < 0.05). Except for the kinematic features of F and Ncyc, which were greater in the standard AMR task, the change in the rest of kinematic features were directed toward the fixed-target AMR task. These findings verified that the fixed-target AMR task elicited larger kinematic changes than did the standard AMR task (e.g., increased lip opening and maximum velocity in the fixed-target task as compared to the standard task). Compared to the healthy controls, participants with ALS had a smaller number of features that were statistically significantly different between the standard and fixed-target AMR task (*p* < 0.05). Similar to healthy controls, the between-task changes of F and Ncyc were directed toward the standard AMR tasks in the ALS group. In addition to F and Ncyc, standard the AMR task elicited larger changes in the kinematic feature of Slp_1 in patients with ALS.

**Figure 3:**
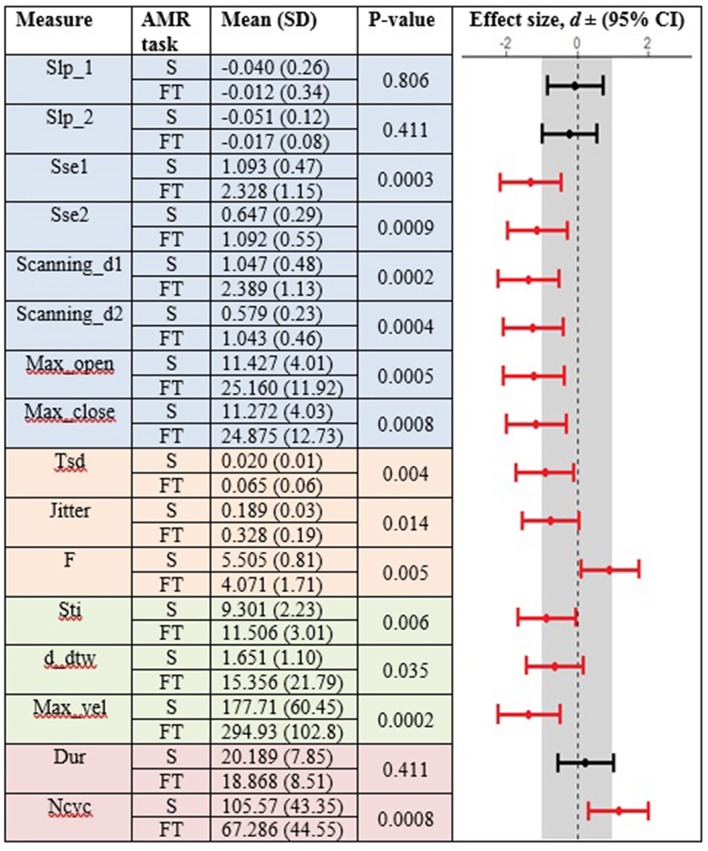
Kinematic features extracted from lip movement during standard (S) and fixed-target (FT) AMR tasks in the healthy control (HC) group.

**Figure 4:**
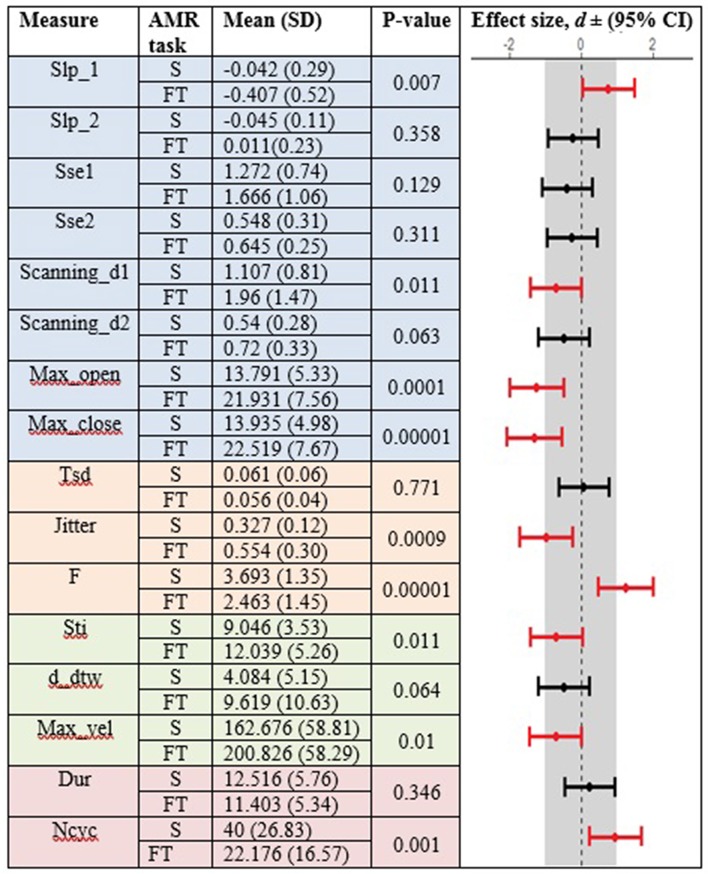
Kinematic features extracted from lip movement during standard (S) and fixed-target (FT) AMR tasks in the ALS group.

Figures 3, 4 display the within-group comparison of standard and fixed-target AMR tasks in healthy controls and participants with ALS, respectively. In each table, the effect sizes are represented by dots at the middle of each horizontal line and each line represents the 95% confidence interval (CI) around the corresponding effect size. The red lines indicate statistically significant comparisons between kinematic measures derived from standard and fixed-target AMR tasks (*p* < 0.05). Horizontal lines deviated to the left indicate the directionality of change toward the fixed-target AMR task and those deviated to the right show the directionality of change toward the standard AMR task.

### Between-Group Comparison of Standard and Fixed-Target AMR Tasks

**Figure 5:**
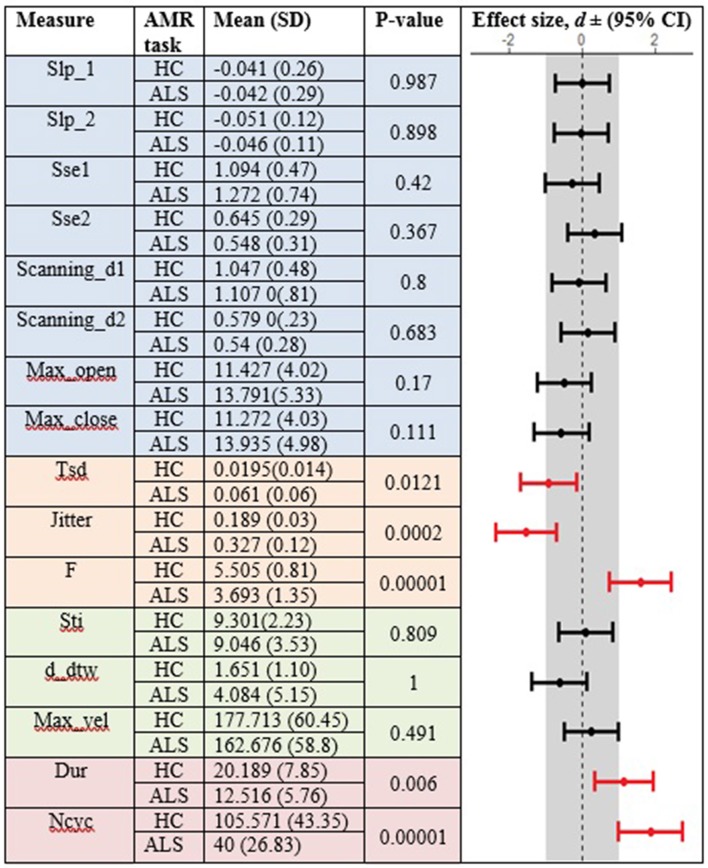
Comparison of kinematic features between the healthy control (HC) and ALS groups obtained during the standard AMR task.

**Figure 6:**
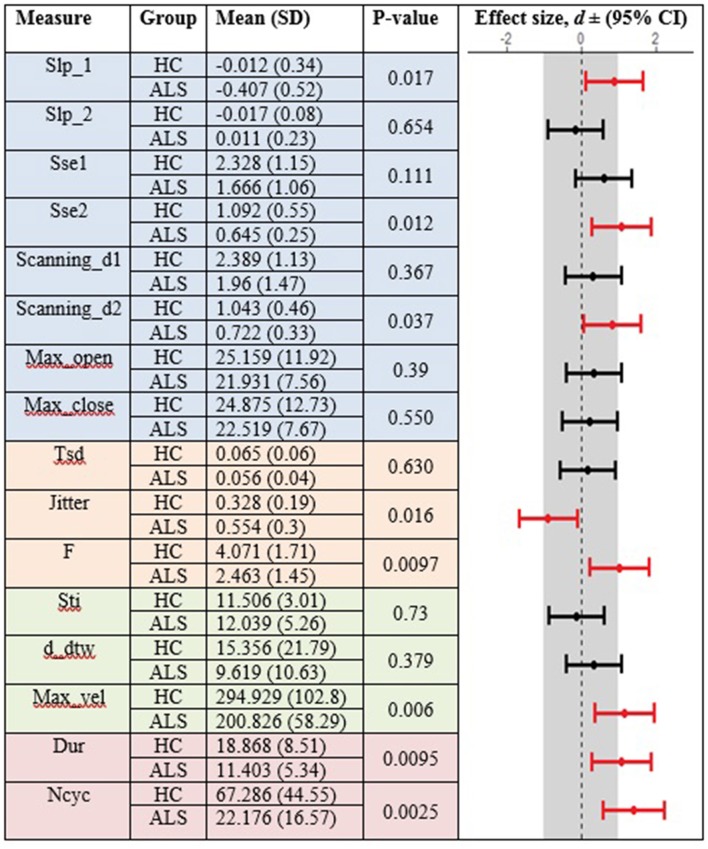
Comparison of kinematic features between the healthy control (HC) and ALS groups obtained during the fixed-target AMR task.

In the standard AMR task, significant group differences were observed for temporal kinematic features (Tsd, Jitter, and F) and for features of overall motor performance (Dur, Ncyc) (*p* < 0.05). The fixed-target AMR task revealed significant between-group differences for spatial kinematic features (Slp_1, Sse2, Scanning d2), spatiotemporal kinematic features (Max_vel), temporal kinematic features (Jitter, F), and features of overall motor performance (Dur, Ncyc) (*p* < 0.05). Figures 5, 6 represent results of between-group comparisons in the standard and fixed-target AMR tasks, respectively. In each table, the effect sizes are represented by dots at the middle of each horizontal line and each line represents the 95% confidence interval (CI) around the corresponding effect size. The red lines indicate statistically significant comparisons between healthy controls and participants with ALS (*p* < 0.05). Horizontal lines deviated to the left indicate the directionality of change toward the ALS group and those deviated to the right show the directionality of change toward healthy controls.

**Figure 7:**
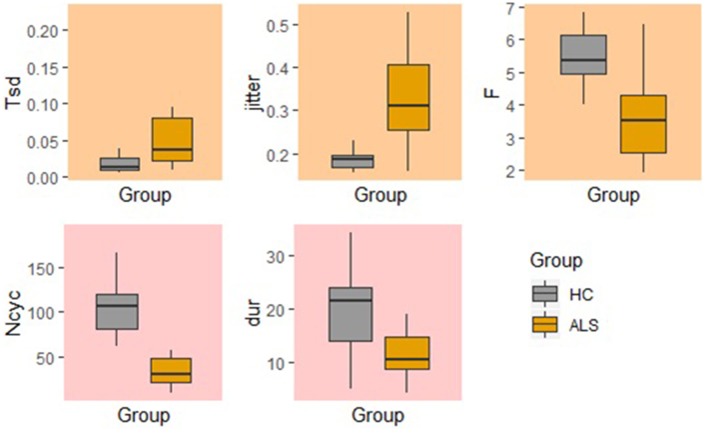
Significant kinematic features that differentiate between the healthy control (HC) and ALS groups in the standard AMR task.

**Figure 8:**
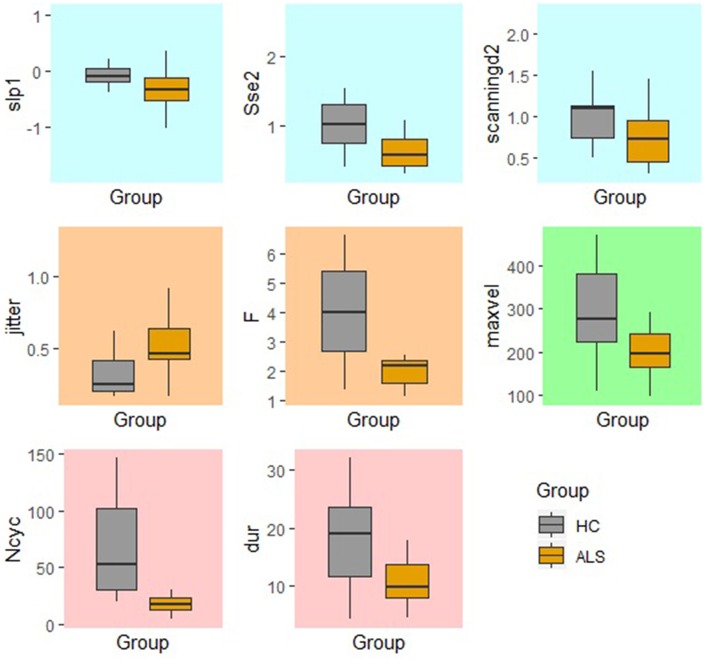
Significant kinematic features that differentiate between the healthy control (HC) and ALS groups in the fixed-target AMR task.

In comparison to the standard AMR task, the fixed-target AMR task yielded higher inter-subject variability in healthy controls. Figures 7, 8 illustrate inter-subject variability within each kinematic feature that induced significant differences between the two groups in the standard and fixed-target AMR tasks, respectively.

### Verification of the Max_vel Kinematic Feature Based on the ALSFRS-R Bulbar Subscores

**Figure 9:**
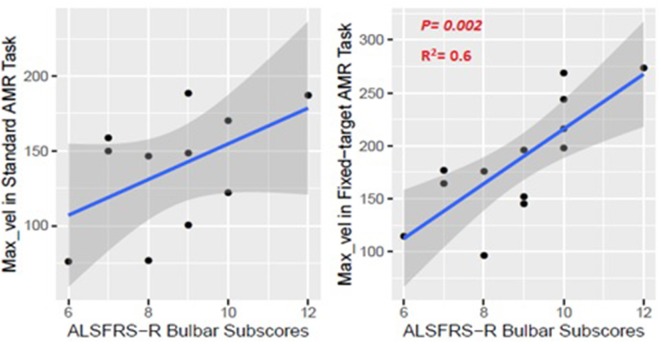
Numerical association between the Max_vel kinematic feature extracted from the lip movement during the AMR tasks (standard and fixed-target) and the ALSFRS-R bulbar subscores.

The kinematic feature of Max_vel extracted from the lip movement during the fixed-target AMR task was able to statistically significantly predict the ALSFRS-R bulbar subscores (*R*^2^ = 0.60, *p* = 0.002) (Figure 9). No statistically significant association was observed between the measure of Max_vel extracted from the lip during the standard AMR task and the ALSFRS-R bulbar subscores (*R*^2^ = 0.25, *p* = 0.25).

## Discussion

In this methodology study, we compared two types of AMR task (i.e., standard and fixed-target) in their ability to test maximum motor performance in healthy individuals and patients with ALS who demonstrated a wide range of severity in terms of intelligibility, speaking rate, and ALSFRS-R total scores and bulbar subscores. We used a semi-automated algorithm to extract kinematic features that probe spatial, temporal, and spatiotemporal properties of lip articulatory movement as well as the overall motor performance. Four main findings of the study are summarized below:

### The Fixed-Target AMR Significantly Increased Motor Demands

Results of this study revealed that, in general, the fixed-target AMR task imposed greater articulatory demands on the motor system relative to the standard AMR task. The fixed-target task was effective in minimizing the truncation of articulatory displacement by eliciting larger articulatory displacements.

In healthy controls, the fixed-target AMR task elicited larger articulatory excursions (Max-open), faster velocities (Max-vel), greater variability in cycle durations (Tsd), larger absolute difference in duration of consecutive cycles (jitter), and increases in indices of spatiotemporal variability (i.e., Sti, D_dtw). Similarly, in the ALS group, spatial and spatiotemporal features indexed by maximum lip opening and closing gestures (Max-open, Max-close), spatiotemporal variability (Sti), and maximum velocity (Max-vel) were significantly different between the two tasks, likely resulting from larger displacements and greater variability in cycle production induced by the fixed-target task. The elicitation of fast movement speeds while maintaining consistent articulatory displacements suggests that the fixed-target AMR task is a more valid test of maximum performance relevant to the standard AMR task. These findings are consistent with those reported by Mefferd et al. (25) in which movement speed was induced by implementing a metronome-paced fixed-target AMR task in order to drive jaw displacement.

As expected, the number of cycles (Ncyc) and frequency of syllable repetitions (F) significantly decreased in the fixed-target AMR task compared to the standard AMR task in both groups. To complete each syllable cycle, the lower jaw traveled a longer distance to reach the target than it did in the standard AMR task, and subsequently, the total number of syllables decreased. Additionally, the increased motor demands of the fixed-target AMR task may have induced fatigue. The steeper negative slope of lip opening (Slp-1) observed in the ALS group is consistent with the presence of fatigue because it suggests a progressive truncation of movement extent across syllable repetitions.

### Reduced Task-Adaptation Is a Prominent Characteristic of Bulbar Motor Involvement in ALS

Between-task comparisons (standard vs. fixed-target AMR) in healthy controls and participants with ALS revealed that speakers with impaired motor systems have a limited capacity to accommodate the higher articulatory demands imposed on the neuromotor system during the fixed-target AMR task. Healthy controls, however, uniformly adjusted articulatory control to accommodate each task, as evidenced by the large effect sizes presented in Figure 3. In contrast to the healthy controls, fewer of the kinematic features differed between tasks in the ALS group. These findings are consistent with our prior work in which individuals at the early stage of ALS (bulbar asymptomatic), who had speaking rate and speech intelligibility within normal limits, exhibited reduced patterns of task adaptation during a fixed-target AMR task (20). Additional research is warranted to determine if the inability to adapt to the demands of a fixed-target AMR task is an early indicator of bulbar motor involvement due to ALS.

### Fixed-Target AMR Tasks Comprehensively Probed Multiple Aspects of Speech Motor Performance

The findings of the current study suggested that the kinematic features of jitter, F, Ncyc, and duration are robust indices of motor performance that can differentiate between the two groups of participants regardless of the type of the AMR task. In the standard AMR task, participants with ALS were significantly different from healthy controls in temporal features (Tsd, Jitter, and F), as well as in features of overall motor performance (Dur, Ncyc). Fixed-target AMR tasks, however, induced significant differences between the two groups in a wider range of kinematic features that included spatial (Slp-1, Sse2, Scanning d2) and spatiotemporal features (Max-vel) along with temporal features (Jitter, F) and features of overall motor performance (Dur, Ncyc). Therefore, while the standard AMR task elicited changes primarily in temporal features, the fixed-target AMR task elicited changes in all aspects of motor performance—primarily in spatial and spatiotemporal features and secondarily in temporal features. Additional work is needed to test the clinical efficacy of the task for early detection, indexing disease severity, and monitoring disease progression.

### Maximum Velocity of Lip Movement During the Fixed-Target AMR Task Was Associated With the ALSFRS-R Bulbar Subscores

The maximum velocity (Max_vel) was associated with the ALSFRS-R bulbar subscore. The observed coefficient of determination suggested a moderate goodness-of-fit relevant to the regression line (*R*^2^ = 0.6), which is acceptable particularly given that ALSFRS-R bulbar subscores are calculated based on patients' self-reports. This finding provides additional support for (1) validity the fixed-target task in eliciting the velocity of movement by preventing articulatory truncation under fast speaking rate condition, and (2) the clinical utility of this measure for staging bulbar motor involvement.

## Limitations of the Study

Several limitations of this study need to be acknowledged. First, because of the small sample size in both groups, conducting Bonferroni corrections to control the familywise error rate could have increased the occurrence of type II error; interpretation of effect sizes of the corresponding comparisons was, therefore, used to support our inferences. Second, because the data used in this study was obtained as part of the protocol for a larger project, the standard AMR task consistently preceded the fixed-target AMR task. Inability to randomize the order of the tasks could have introduced potential confounds. Third, due to the small sample size, sub-analyses considering differential subsystem involvement on speech performance of patients with ALS were not performed. Additionally, the neuropsychological profile of the patients with ALS was unknown as cognitive data were not available for all patients with ALS. More studies with larger sample sizes, controlled jaw-to-target distance, and known neuropsychological and subsystem (e.g., respiratory) profiles of patients with ALS are warranted to substantiate the findings of this study.

## Conclusions

The current study provided empirical evidence in support of the effectiveness of fixed-target AMR tasks in comparison to standard AMR tasks to prevent speakers from truncation of articulatory displacement rather than speeding articulatory movements under fast speaking conditions. Findings demonstrated that although the fixed-target AMR task elicited larger articulatory displacements and larger temporal and spatiotemporal variabilities in healthy controls, participants with ALS exhibited reduced capability to make the required articulatory adjustments due to underlying neurologic deficits. Reduced task adaptation was considered as an indicator of bulbar motor involvement that could be used clinically for monitoring neuromotor performance across disease progression. Additionally, while the standard AMR task predominantly relied on temporal variabilities to differentiate between healthy controls and participants with ALS, the fixed-target AMR task was able to discriminate between the two groups using spatial and spatiotemporal kinematic features in addition to the temporal features. These findings suggest that fixed-target AMR tasks can facilitate a multi-faceted evaluation of motor capacity by challenging the neuromotor system in both spatial and temporal domains of speech motor performance. For a comprehensive neuromotor evaluation, however, the combination of these two AMR tasks is preferable as each predominately probes different aspects of motor performance. Finally, the lip maximum velocity during the fixed-target AMR task showed acceptable association with the ALSFRS-R bulbar subscores.

Findings from this study have several clinical implications. First, results can help clinicians better understand the shortcomings of the traditional AMR task and may be encouraged to use the additional fixed-target AMR task for the assessment of neuromuscular integrity of the orofacial system in patients with ALS. Given that the administration of the fixed-target AMR task does not require sophisticated instrumentation or a high-tech laboratory setting, patients can be easily instructed to perform the task during a clinical evaluation. The algorithm to extract the kinematic data from the lip movement can, in the future, be embedded into a software application on a laptop computer, mobile, or tablet, where simultaneous recording of the audio and video signals is possible. Acoustic and kinematic data obtained from the input signals can facilitate clinical decision making and allow clinicians to better monitor disease progression.

## Data Availability Statement

The datasets generated for this study are available on request to the corresponding author.

## Ethics Statement

The studies involving human participants were reviewed and approved by PARTNER's health care IRB. The patients/participants provided their written informed consent to participate in this study.

## Author Contributions

ME had major role in study conceptualization and design, data analyses, interpretation of the findings, and writing the manuscript. KS contributed to the data collection and interpretation of the findings, reviewed the manuscript, and provided the feedback. AM contributed to the interpretation of the findings, reviewed the manuscript, and provided the feedback. PR contributed to the analysis tool and interpretation of the findings, reviewed the manuscript, and provided the feedback. JB reviewed the manuscript and provided the feedback. YY contributed to the interpretation of the findings, reviewed the manuscript, and provided the feedback. JG had major role in the study conceptualization and design, interpretation of the findings, and manuscript preparation.

### Conflict of Interest

The authors declare that the research was conducted in the absence of any commercial or financial relationships that could be construed as a potential conflict of interest.
